# Ultrasound-guided supraclavicular block versus Bier block for emergency reduction of upper limb injuries: a protocol for an open-label, non-inferiority, randomised controlled trial

**DOI:** 10.1186/s13063-023-07403-z

**Published:** 2023-05-30

**Authors:** Henry Tsao, Christopher Tang, Mark Trembath, Philip Jones, Peter J. Snelling

**Affiliations:** 1 Emergency Department, Redland Hospital, Cleveland, QLD Australia; 2 School of Medicine, The University of Queensland, Herston, QLD Australia; 3 Department of Anaesthetics, Royal Brisbane and Women’s Hospital, Brisbane, QLD Australia; 4 Department of Emergency Medicine, Gold Coast University Hospital, Southport, QLD Australia; 5 School of Medicine and Dentistry, Griffith University, Southport, QLD Australia; 6 Sonography Innovation and Research Group (Sonar Group), Southport, QLD Australia

**Keywords:** Supraclavicular block, Nerve block, Bier block, Regional anaesthesia, Emergency medicine, Randomised controlled trial, Ultrasonography, Upper limb injury, Pain

## Abstract

**Background:**

Closed reduction of upper limb fractures and/or dislocations are common in the emergency department (ED), which involves physically re-aligning the injured part prior to immobilisation. As this is painful, numerous techniques are available to provide regional anaesthesia to ensure patient tolerance. A Bier block (BB) is typically performed as part of routine care. An alternative technique gaining interest in the ED is ultrasound-guided supraclavicular block (UGSCB), which involves injecting local anaesthetic around the brachial plexus at the supraclavicular fossa under ultrasound guidance. It is unclear whether UGSCB is effective and safe when performed in the ED.

**Methods:**

SUPERB (SUPraclavicular block for Emergency Reduction versus Bier block) is a prospective open-label, non-inferiority randomised controlled trial that compares the effectiveness of UGSCB versus BB for closed reduction of upper limb fractures and/or dislocations. Adult patients presenting with an upper limb fracture and/or dislocation that requires emergent closed reduction in the ED will be eligible. After screening, participants will be randomised to either UGSCB or BB. Once regional anaesthesia is obtained, closed reduction of the injured part will be performed and appropriately immobilised. The primary outcome is maximal pain experienced during closed reduction measured via a visual analogue scale (VAS). Secondary outcomes include baseline and post-reduction pain, patient satisfaction, total opioid requirement in ED, ED length of stay, adverse events and regional anaesthesia failure.

**Discussion:**

Existing evidence suggests UGSCB is effective when performed in the operating theatre by trained anaesthetists. SUPERB will be the first randomised controlled trial to elucidate the effectiveness and safety of UGSCB in the ED. The findings have the potential to provide an alternative safe and effective option for the management of upper extremity emergencies in the ED.

**Trial registration:**

This trial has been registered on 21 October 2022 with Australia and New Zealand Clinical Trials Registry (ACTRN12622001356752).

## Administrative information

Note: the numbers in curly brackets in this protocol refer to SPIRIT checklist item numbers. The order of the items has been modified to group similar items (see http://www.equator-network.org/reporting-guidelines/spirit-2013-statement-defining-standard-protocol-items-for-clinical-trials/).

**Table:** 

Title {1}	Ultrasound-guided supraclavicular block for emergency reductions of upper limb injuries versus Bier block: a study protocol for an open-label, non-inferiority, randomised controlled trial
Trial registration {2a and 2b}.	Trial identifier: ACTRN12622001356752 Registry name: Australia and New Zealand Clinical Trials Registry (ANZCTR) Date of registration: 21/10/2022 URL: https://www.anzctr.org.au/Trial/Registration/TrialReview.aspx?id=384836&isReview=true All items from the WHO Trial Registration Dataset can be found on the ANZCTR.
Protocol version {3}	06/03/2023; Version 1.0
Funding {4}	The research is funded by a project grant from the Metro South Health Study, Education and Research Trust Account and Research Support Scheme (RSS_2023_102), and an emerge grant from Emergency Medicine Foundation (EMEG-030R38-2022-TSAO).
Author details {5a}	Henry Tsao^1,2^, Christopher Tang^1,2^, Mark Trembath^2,3^, Philip Jones^4^, Peter J. Snelling^2,4–6^ Affiliations: 1. Emergency Department, Redland Hospital, Cleveland, Queensland, Australia. 2. School of Medicine, The University of Queensland, Herston, Queensland, Australia. 3. Department of Anaesthetics, Royal Brisbane and Women’s Hospital, Brisbane, Queensland, Australia. 4. Department of Emergency Medicine, Gold Coast University Hospital, Southport, Queensland, Australia 5. School of Medicine and Dentistry, Griffith University, Southport, Queensland, Australia 6. Sonography Innovation and Research Group (Sonar Group), Southport, Queensland, Australia
Name and contact information for the trial sponsor {5b}	Manager, Integrity and Compliance Metro South Research L7, 37 Kent Street Woolloongabba, QLD 4102 Telephone: + 61 7 3443 8050 Email: MSH-RGO@health.qld.gov.au
Role of sponsor {5c}	The sponsor is responsible for ensuring appropriate approvals are obtained prior to commencing the clinical trial and that the research protocol is adhered to during the clinical trial. The sponsor has no role in the study design, data collection or publication.

## Introduction

### Background and rationale {6a}

Closed reduction of upper limb fractures and/or dislocations are commonly performed in the emergency department (ED). This involves physically manipulating the injured part to restore anatomical alignment prior to immobilisation. As this is a painful procedure, there are numerous techniques available to provide regional anaesthesia to ensure patient comfort and tolerance. Bier block (BB) has traditionally been advocated as the preferred technique in the ED, where intravenous (IV) injection of local anaesthetic is performed to the injured limb with a tourniquet applied proximally to prevent systemic spread [1]. This anaesthetises the upper limb distal to the tourniquet. BB is considered routine care across many Australian EDs [2], but is often associated with discomfort from the tourniquet pressure, limited by numerous contraindications, and there can be difficulty obtaining IV access on the injured limb due to swelling and/or bruising [3]. Procedural sedation with IV agents is an alternative option if there are contraindications to BB, but this can be resource intensive with risk of adverse effects, particularly in patients with multiple co-morbidities [4].

An alternative regional anaesthetic technique gaining traction in the ED is the ultrasound-guided supraclavicular block (UGSCB; [5]). Using ultrasound, the brachial plexus that innervates the upper limb at the supraclavicular fossa is identified. A needle is inserted to infiltrate local anaesthetic around these nerves under direct visualisation [6], which results in anaesthesia of the ipsilateral arm. UGSCB has numerous advantages in the ED including familiarity of the anatomy of the supraclavicular region amongst emergency physicians due to ultrasound-guided central venous line placement, its relative ease of being performed, potential for prolonged analgesia and relatively low incidence of complications [7, 8]. Evidence supporting the effectiveness of UGSCB has been performed predominantly in the operating theatre by anaesthetists for surgical management of upper limb injuries [9, 10]. However, it remains unclear whether UGSCB, when performed by emergency physicians in the ED, is an effective and safe alternative for providing regional anaesthesia for closed reduction of upper limb fractures or dislocations. Therefore, the objective of this trial is to evaluate whether the pain associated with limb reduction after UGSCB is non-inferior in comparison to that after BB in the ED, using a randomised controlled trial design.

### Objectives {7}

The primary objective of the SUPraclavicular block for Emergency Reduction of upper limb injuries versus Bier block (SUPERB) trial is to determine the effectiveness of UGSCB for providing regional anaesthesia for closed reduction of upper limb fractures and/or dislocations in the ED compared to BB, with a non-inferiority hypothesis for patient reported procedural pain. The secondary objectives are to assess post-reduction pain, patient satisfaction, total opioid requirement in ED, ED length of stay, adverse events and regional anaesthesia failure following UGSCB with comparison to BB.

### Trial design {8}

SUPERB is an open-label, single-site, non-inferiority randomised controlled trial (RCT), with a 1:1 allocation ratio. The protocol adheres to the Standard Protocol Items: Recommendations for Interventional Trials (SPIRIT) Statement.

## Methods: Participants, interventions and outcomes

### Study setting {9}

SUPERB will be conducted at Redland Hospital ED, which is an urban district hospital in Brisbane with an annual census of ~ 55,000 presentations. There is limited on-site orthopaedic service during the weekdays and no inpatient orthopaedic service after hours or on weekends (with patients either managed in ED or transferred to the nearest centre during these times). There are ~ 100 upper limb reductions performed in our ED annually.

### Eligibility criteria {10}

Potentially eligible participants will be approached and invited to participate in the study by the research investigator, research assistant or treating clinician. The inclusion and exclusion criteria are outlined in Table 1, with the main criteria being adults presenting to ED with an upper limb fracture and/or dislocation requiring closed reduction with no contraindication to either method of regional anaesthesia. Patients who are screened and excluded will be noted, including the comparison of contraindications due to either technique.

**Table 1:** Inclusion and exclusion criteria

Inclusion criteria	Exclusion criteria
• Aged 18 or older • Capacity to provide informed written consent • Upper limb fracture and/dislocation requiring ED closed reduction and immobilisation	• Refusal to consent • Local anaesthetic allergy • Open fractures, unstable fracture and/or dislocations that require urgent surgical fixation • Pregnancy • Anticoagulation • Chronic lung disease (e.g., chronic obstructive pulmonary disease or unstable asthma) • Skin infection over the supraclavicular site of injection • Previous surgery or radiation therapy to the supraclavicular region • Severe hypertension (> 160 mmHg) • Compartment syndrome • Congenital or idiopathic methaemoglobinaemia • Sickle cell disease • Peripheral vascular disease • Peripheral neuropathy • Cardiac conduction abnormality

### Who will take informed consent? {26a}

Once patients are deemed eligible for the study (after confirmation of forearm or elbow fracture/dislocations on X-ray and satisfying the inclusion and exclusion criteria), they are approached to participate in the trial by the treating clinician or a member of the research team. An information sheet outlining the research study will be provided. Written informed consent will be obtained by a member of the research team (research investigator or assistant) or the treating emergency physician.

### Additional consent provisions for collection and use of participant data and biological specimens {26b}

There are no plans for ancillary studies in the future using the data collected in this trial.

## Interventions

### Explanation for the choice of comparators {6b}

#### Bier block

In many EDs, BB remains the standard option for providing regional anaesthesia to allow for painful procedures involving the upper limb to be undertaken [2]. A BB will be performed by a qualified emergency physician (i.e. certified by the Australasian College for Emergency Medicine). The participant will be positioned in semi-reclined sitting. Monitoring will include continuous pulse oximetry, five-minute interval blood pressure on an unaffected limb and three-lead electrocardiography (ECG). An intravenous cannula (IVC) will be inserted at the wrist/hand or forearm of the injured limb. A second IVC will be available on the unaffected limb. The BB cuff (AT4, AneticAid, West Yorkshire, United Kingdom) will be applied at the proximal upper limb. The arm is then elevated for five minutes to enhance venous drainage, before the cuff is inflated to 100 mmHg above the patient’s systolic blood pressure (to a maximum of 250 mmHg). Prilocaine (0.5%, 5 mg/kg to maximum of 250 mg) or lignocaine (0.5%, 3 mg/kg to maximum of 200 mg) will be injected via the IVC on the affected side. Adequacy of the block will be assessed by the treating clinician prior to closed reduction of the injured part and immobilisation. The BB cuff is then slowly released after 30 min.

### Intervention description {11a}

#### Ultrasound-guided supraclavicular block

**Fig. 1:**
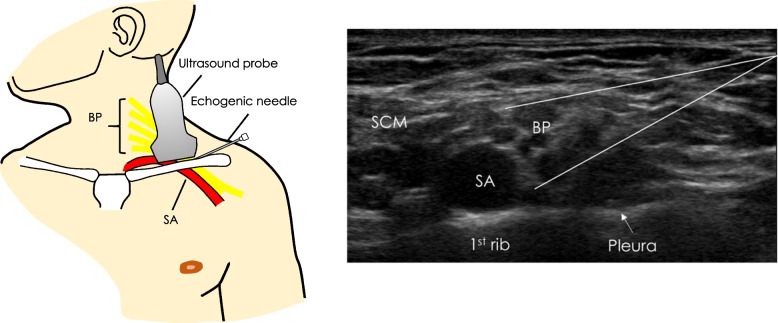
Ultrasound-guided supraclavicular block (UGSCB): Left panel illustrating ultrasound probe and needle placement. Right panel showing ultrasound anatomy of the supraclavicular fossa, with superimposed needle trajectories for UGSCB. Abbreviations: BP-Brachial plexus, SA-Subclavian artery, SCM-sternocleidomastoid

UGSCB will be performed by emergency physicians who have obtained competency through a one-hour training workshop and performance of at least two UGSCB under direct supervision of an emergency physician competent in the procedure. To perform UGSCB, participants will be in a semi-reclined sitting position, with their head turned away from the side to be block. Monitoring will include continuous pulse oximetry, five minutely blood pressure on the unaffected limb and three-lead ECG. Aseptic technique will be adhered to including sterile gloves, sterile ultrasound probe cover and sterile gel. The supraclavicular region will be prepared with chlorhexidine and draped. Ultrasound imaging of the supraclavicular fossa will be undertaken using a 12 MHz linear probe (Sonosite X-Porte, Fujifilm, Washington USA) or equivalent. The brachial plexus will be identified, and its relation to the subclavian artery, pleura and first rib. Colour Doppler is used to identify and avoid blood vessels. Local anaesthetic (2–3 ml 1% xylocaine + adrenaline) will be injected using a 25G needle to anaesthetise the overlying skin. A 21 G × 110 mm echogenic needle (Sonoplex II, Pajunk, Geisingen Germany) attached to a 20 ml syringe will be inserted under ultrasound guidance to deposit local anaesthetic (20 ml of 0.75% ropivacaine) inferior and superior to the brachial plexus bundle (Fig. 1). Evidence suggests that a two-injection technique is associated with better regional anaesthesia compared to a single-injection technique [11]. Adequacy of regional block will be assessed by the treating clinician prior to closed reduction of the injured part and immobilisation.

### Criteria for discontinuing or modifying allocated interventions {11b}

Should the participant demonstrate inadequate anaesthesia from either UGSCB or BB, as evidenced by the inability to tolerate closed reduction of the injured part, the treating clinician can choose an additional or alternative method that will allow the participant to undergo safe and comfortable closed reduction of the injured limb. The choice of technique will be at the discretion of the treating clinician in consultation with the patient, and may include the use of inhaled nitrous, performing the alternative trial intervention (BB or UGSCB), IV procedural sedation, or abandonment and referral to a centre with theatre access for orthopaedic procedures.

### Strategies to improve adherence to interventions {11c}

Treating clinicians will be educated on the research trial and supported by the research investigators and assistants. A checklist will be utilised to maximise adherence to the intervention protocols.

### Relevant concomitant care permitted or prohibited during the trial {11d}

Participants can receive concomitant analgesia including inhaled nitrous, oral and/or IV analgesia during the study as clinically indicated. The type and/or total dose of analgesia received in the ED will be recorded and compared between the two study groups.

### Provisions for post-trial care {30}

Following closed reduction, the injured part will be typically immobilised with plaster of paris with repeat x-rays performed to assess anatomical alignment of the injured part. After adequacy of reduction, the participant will be discharged from the ED with orthopaedic follow-up within approximately one week for review and ongoing management. In the event where there is inadequate reduction or neurovascular injury requiring urgent treatment, participants will be managed by the acute orthopaedic service. In the event where a participant experiences adverse effects from either BB or UGSCB, they will be managed appropriately by the treating emergency physician in the ED. The type and severity will be compared between groups.

### Outcomes {12}

The primary outcome of the study will be the maximal level of pain experienced during closed reduction of the injured part, as reported by the participant on a 10 cm visual analogue scale (VAS) immediately after completion of the closed reduction procedure. The patient will be asked by the research investigator, research assistant or treating clinician to mark their pain on a VAS scale printed on paper anchored with “no pain” at 0 cm and “pain as bad as it could possibly be” at 10 cm. The 10 cm VAS has been demonstrated previously to be valid and reliable for participant-reported pain [12].

Secondary outcome measures will include:

- Baseline pain: Participant reported pain will be measured using a 10 cm VAS immediately prior to the commencement of either UGSCB or BB. Any analgesia administered prior to the procedure will be compared between groups to ensure that they are similar.
- Pain at one hour post-procedure: Participant reported pain at one hour post procedure (or just prior to discharge if under an hour) will be measured using a 10 cm VAS.
- Patient satisfaction will be measured using a 10 cm VAS satisfaction scale anchored with “extremely dissatisfied” at 0 cm and “extremely satisfied” at 10 cm. This will be measured one hour post procedure (or just prior to discharge if under an hour). The patient satisfaction VAS has been validated to be a reliable tool to evaluate patient satisfaction [13].
- ED length of stay: the time (minutes) from triage to readiness to be discharged from the ED, will be obtained from the electronic medical record.
- Analgesia: The total opioid use including those given by the ambulance service pre-hospital and those administered in the ED will be recorded. Total opioid use will be converted to oral morphine milligram equivalent using Australian and New Zealand College of Anaesthetists Faculty of Pain Medicine opioid calculator (http://www.opioidcalculator.com.au).
- Management: The number of sedations and techniques required in ED, the number of inpatient transfers, and the number of manipulations or operations at another hospital will be compared between groups.
- Adverse events/complications: Any adverse events experienced by the participant or noted by the treating clinician will be recorded. We will also contact the participant via email 24–72 h after discharge from the ED to evaluate any adverse events post-discharge.
- Adjunct therapies or treatment failure for UGSCB and BB will also be compared between the two groups.

### Participant timeline {13}

Table 2 Shows a schematic of the participant timeline.

**Table 2:** Timeline for data collection (SPIRIT Figure)

	Screening	Allocation	Post-allocation			Close-out	
TIMEPOINT	Pre-intervention		Intervention	Closed reduction & immobilisation	Post-intervention	Discharge from ED	Post Discharge from ED
ENROLMENT:							
Eligibility screen	X						
Informed consent	X						
Participant demographics	X						
Randomisation		X					
INTERVENTIONS:							
*UGSCB*			X				
*BB*			X				
Closed reduction & immobilisation				X			
ASSESSMENTS:							
Pain VAS during closed reduction^a^				X			
Baseline Pain VAS	X						
Pain VAS prior to discharge					X		
Patient satisfaction VAS					X		
ED length of stay						X	
Opioid use						X	
Adverse events or complications						X	
Adjunct therapy or treatment failures						X	
Number of sedations and techniques used in ED						X	
Number of inpatient transfers, subsequent sedations, or operations							X

*Abbreviations*: *UGSCB* Ultrasound-guided supraclavicular block, *BB* Bier block, *VAS* Visual analogue scale, *ED* Emergency department

^a^Primary outcome

### Sample size {14}

Sample size calculations were based upon a hypothesis of non-inferiority of UGSCB compared to BB for the primary outcome measure of maximal pain during closed reduction. Sample size calculations were based upon approximation to the normal distribution [14] using previous pain VAS scores reported by Kukreja et al. [9] and Beck et al. [15]. A non-inferiority margin of 20 mm was selected for power calculation. This was based on a previous study [15] that compared Bier block to a periosteal block (i.e., injection of local anaesthetic at the fracture site) for closed reduction of distal radius fractures. That study showed median pain VAS during closed reduction was 5 mm for Bier block and 26 mm for periosteal block (i.e., difference of 21 mm on pain VAS, which was rounded to 20 mm), but both techniques provided adequate anesthesia for tolerance of closed reduction procedure. Thus, a non-inferiority margin of 20 mm difference in mean VAS score was selected for the trial.

Assuming a non-inferiority margin of 20 mm difference in the mean VAS pain score, expected standard deviation of 30 mm [9], alpha 0.025 and a power of 80%, we would require primary outcome data for 72 participants. Allowing for an attrition rate of 5%, we estimate that we would require a total sample size of 76 participants for the study (i.e. 38 participants per group).

### Recruitment {15}

To maximise participant recruitment, we will hold several training sessions to have emergency physicians trained in both UGSCB and BB available at the time of patient recruitment and randomisation. We will also educate all ED staff regarding the eligibility criteria for the trial. Coverage will be available during day and evening shifts, with variable coverage at night depending on the senior doctor rostered.

## Assignment of interventions: allocation

### Sequence generation {16a}

Randomisation will be concealed by using the Griffith Randomisation Service (http://randomisation.griffith.edu.au/) to randomise participants to either UGSCB or BB. This involves a centralised, computer-generated algorithm with block randomisation performed in a 1:1 ratio to ensure equal numbers in each group.

### Concealment mechanism {16b}

This is already described in 16a.

### Implementation {16c}

Participants eligible for the research trial will be enrolled by either the research investigator, research assistant or treating clinician. Allocation of participants to either UGSCB or BB will be performed using the Griffith Randomisation Service.

## Assignment of interventions: blinding

### Who will be blinded {17a}

The trial will be conducted as an open-label study, with participants and treating clinicians aware of the group allocation. Outcome assessors and data analysts will be masked to the group allocation when analysing the primary and secondary outcomes.

### Procedure for unblinding if needed {17b}

There will be no unblinding required until the completion of the trial.

## Data collection and management

### Plans for assessment and collection of outcomes {18a}

Research staff or trained clinicians will enter the data onto a paper based clinical research form, which was designed by the research team. Once data collection is completed for each participant, the data will then be entered into an electronic database (REDCap), which has built-in checks to ensure that the data collected is complete. The database which will be later assessed by a research member blinded to the group allocation.

### Plans to promote participant retention and complete follow-up {18b}

The collection of primary and secondary outcome data will occur before, during and after the allocated intervention and closed reduction during the index ED presentation, as well as from routine medical records obtained during that presentation. As data is obtained during the same clinical event as participant recruitment, rates of attrition are expected to be low. Should a participant elect to withdraw from the study, no further additional information will be collected, although non-identifiable data such as pain scores already collected will be retained to ensure the result of the study can be measured.

### Data management {19}

Data generated by the project staff will be stored in a secure password protected internet-based database (REDCap) with access only by the research investigators involved in the study. Paper-based data will be kept in a locked cabinet in the office of the principal investigator. We will maintain the data for at least 15 years as recommended by the National Health and Medical Research Council of Australia. After this period of time, digital data will be destroyed by deleting the database and paper records will be shredded.

### Confidentiality {27}

Each participant will be allocated a unique identification code in order to protect their identity. Any data collected will then be recorded with respect to this code. No individual data will be reported in the trial.

### Plans for collection, laboratory evaluation and storage of biological specimens for genetic or molecular analysis in this trial/future use {33}

This study does not involve collection of biological specimens.

## Statistical methods

### Statistical methods for primary and secondary outcomes {20a}

Group data will be used for data and statistical analysis. Data will be summarised as mean (standard deviation) or median (interquartile range) for continuous variables, depending on the normality of their distribution, and as frequency (percentage) for categorical variables. The primary outcome of pain intensity during the procedure will be assessed as a non-inferiority hypothesis using data from the 10 cm VAS tool. The between-group difference in pain intensity will be assessed using linear regression modelling with group allocation (UGSCB vs BB) included as a main effect. A conclusion of non-inferiority will be determined if the 95% confidence interval for the between-group difference in mean maximal pain is entirely below the non-inferiority margin. Analysis will be performed on an intention-to-treat basis, with a per-protocol analysis of the primary outcome also included as a key secondary finding.

For analysis of other secondary outcomes, linear regression modelling will be used to compare continuous outcome data, including between-group difference in patient satisfaction on the 10 cm VAS satisfaction scale and total opiate dose in mg of morphine equivalent. Difference in median procedural time and ED length of stay will be compared using median regression. Odds of dichotomous outcomes will be compared using logistic regression modelling. Formal adjustment for multiplicity will not be performed. Analysis will be performed on Stata (StataCorp, Texas USA), v14.2 or later.

### Interim analyses {21b}

There are no plans to perform an interim analysis or stopping guidelines.

### Methods for additional analyses (e.g. subgroup analyses) {20b}

As yet, there is no plan to perform subgroup or post-hoc statistical analysis.

### Methods in analysis to handle protocol non-adherence and any statistical methods to handle missing data {20c}

The primary analysis will be conducted on the modified Intention-to-Treat population, with outcome data for all participants analysed according to their random group allocation, regardless of the actual intervention received (i.e., Bier block or UGSCB) or the occurrence of protocol deviations. Notably, only participants with complete primary outcome data will be included. As Intention-to-Treat analysis may bias towards a finding of no effect, Per-Protocol analysis will also be performed and reported alongside the primary analysis. The Per-Protocol population will consist of all participants who received their allocated intervention and who had no protocol deviation expected to affect the efficacy of regional anaesthesia and analgesia.

The primary outcome will be analysed using complete case data. Missing data will be reported but not imputed. If the proportion of missing data is non-negligible, multiple imputation of missing data will be performed.

### Plans to give access to the full protocol, participant level-data and statistical code {31c}

Data associated with published work will be available upon reasonable request. Should this occur, only de-identified data will be made available to protect participant confidentiality.

## Oversight and monitoring

### Composition of the coordinating centre and trial steering committee {5d}

The coordinating centre is the ED at Redland Hospital in Brisbane, Australia. The trial steering committee consists of the principal investigator (HT) and co-investigations (CT, MT, PJ and PS), who are responsible for participant recruitment, execution of the research protocol, data collection and entry. All members of the trial steering committee are Good Clinical Practice certified.

### Composition of the data monitoring committee, its role and reporting structure {21a}

The Research Governance Office (RGO) of Metro South Hospital and Health Service will ensure the safety of the trial participants by monitoring the ethical conduct of the research study, ensuring that the trial is conducted according to the protocol and that data are collected appropriately.

### Adverse event reporting and harms {22}

Potential harm from UGSCB and BB are listed in Table 3.

**Table 3:** Potential harm/adverse effects

UGSCB	BB
Pain during injection and bruising	Pain during insertion intravenous catheter
Infection	Discomfort of cuff inflation
Injury to major blood vessels or nerves	Infection
Puncture of the lungs	Local bruising
Respiratory distress from phrenic nerve paralysis	Methaemoglobinaemia
Local anaesthetic toxicity	Local anaesthetic toxicity

*UGSCB* Ultrasound-guided supraclavicular block, *BB* Bier block

The occurrence of potential adverse events will be systematically checked from a list and prospectively recorded on the clinical research form. Additionally, other adverse events will be captured from the open-ended question “Were there any other adverse events not listed?” All harms encountered in the trial will be reported in the final report. The principal investigator will report any serious events to the institutional HREC within 72 h of the notice.

### Frequency and plans for auditing trial conduct {23}

Annual progress reports will be submitted to the RGO and the institutional Human Research Ethics Committee (HREC) each year the project is active, and a final report will be submitted on completion of the study. This will include details on trial status, adverse events, complaints and any protocol deviations.

### Plans for communicating important protocol amendments to relevant parties (e.g. trial participants, ethical committees) {25}

Amendments to the research protocol including those that may affect the ongoing ethical acceptability of a protocol will be submitted to the institutional HREC for approval prior to implementation. If approved, changes to the protocol will be communicated through email to the research investigators and treating clinicians involved.

### Dissemination plans {31a}

The findings of the SUPERB study will be disseminated through peer-reviewed publication(s) and academic conference presentations.

## Discussion

The SUPERB trial is novel and could have significant findings that benefit patients and the health care system. This will be the first randomised controlled trial to elucidate the effectiveness and safety of UGSCB compared to BB for the closed reduction of upper limb injuries in the ED. Previous evidence for the efficacy of UGSCB predominantly arise from trials performed by trained anaesthetists in an operating theatre setting [9, 10]. Existing evidence from an ED perspective arise from two case series [7, 16] and a small prospective study [8]. Notably, Stone et al. [8] published a small prospective study including 12 subjects that showed UGSCB was effective and less time consuming when compared to procedural sedation for management of upper limb injuries. Findings from the SUPERB trial could shape future choice of management of upper extremity emergencies.

Further, we anticipate that this research will foster interest in future studies to examine other regional block procedures in the ED, which is rapidly becoming an emerging niche. From the patient perspective, UGSCB may provide additional benefits such as medium-term analgesia, reduction of opioid requirements, and the potential to enhance patient satisfaction. From a departmental/systems perspective, there is potential for UGSCB to reduce overall emergency length of stay, which may facilitate ED patient flow.

## Trial status

The current protocol is version 1.0 dated 06 March 2023, being the final version as approved by HREC and prior to the first participant being recruited. Participant recruitment is anticipated to start in April 2023, with recruitment to be completed by April 2024.

## Data Availability

The data generated by the current study protocol can be accessed by all listed authors. Data will be available from the principal investigator upon reasonable request.

## Abbreviations

BB: Bier block
ECG: Electrocardiography
ED: Emergency Department
HREC: Human Research Ethics Committee
IVC: Intravenous Cannula
RGO: Research governance office
UGSCB: Ultrasound-guided supraclavicular block
VAS: Visual analogue scale
